# Autistic Spectrum Disorder Detection and Structural Biomarker Identification Using Self-Attention Model and Individual-Level Morphological Covariance Brain Networks

**DOI:** 10.3389/fnins.2021.756868

**Published:** 2021-10-08

**Authors:** Zhengning Wang, Dawei Peng, Yongbin Shang, Jingjing Gao

**Affiliations:** School of Information and Communication Engineering, University of Electronic Science and Technology of China, Chengdu, China

**Keywords:** autism spectrum disorder, individual morphological covariance brain networks, self-attention based neural networks, deep learning, biomarker

## Abstract

Autism spectrum disorder (ASD) is a range of neurodevelopmental disorders, which brings enormous burdens to the families of patients and society. However, due to the lack of representation of variance for diseases and the absence of biomarkers for diagnosis, the early detection and intervention of ASD are remarkably challenging. In this study, we proposed a self-attention deep learning framework based on the transformer model on structural MR images from the ABIDE consortium to classify ASD patients from normal controls and simultaneously identify the structural biomarkers. In our work, the individual structural covariance networks are used to perform ASD/NC classification *via* a self-attention deep learning framework, instead of the original structural MR data, to take full advantage of the coordination patterns of morphological features between brain regions. The self-attention deep learning framework based on the transformer model can extract both local and global information from the input data, making it more suitable for the brain network data than the CNN- structural model. Meanwhile, the potential diagnosis structural biomarkers are identified by the self-attention coefficients map. The experimental results showed that our proposed method outperforms most of the current methods for classifying ASD patients with the ABIDE data and achieves a classification accuracy of 72.5% across different sites. Furthermore, the potential diagnosis biomarkers were found mainly located in the prefrontal cortex, temporal cortex, and cerebellum, which may be treated as the early biomarkers for the ASD diagnosis. Our study demonstrated that the self-attention deep learning framework is an effective way to diagnose ASD and establish the potential biomarkers for ASD.

## Introduction

Autism spectrum disorder (ASD) is a developmental disability that can affect significant communications, behavior, and social interactions. The term “spectrum” in ASD is because of the variation in the type and severity of symptoms people experience. The main symptoms of ASD are abnormal emotional regulation and social interaction, limited interest, repetitive behavior, and hypo- or hyper reactivity to sensory stimuli (Guze, 1995). Symptoms will hurt their ability to function properly in school, work, and other areas of life. ASD has caused a severe burden on patients and their families. Therefore, early diagnosis and intervention of ASD are critical. However, the current clinical diagnosis of ASD is mainly based on the doctor’s subjective scale assessment and lacks objective diagnostic methods. The diagnosis based on medical images, especially MRI images, has a certain degree of objectivity, but lacks credible imaging markers. Therefore, objective imaging-based diagnosis of ASD and the provision of reliable imaging markers are significant research trends.

Existing ASD diagnosis methods on structural MRI images are mainly traditional machine learning methods. The handcrafted features in these methods are extracted from morphological structure, such as the cortical thickness of brain gray matter and other geometric features at each cerebral vertex (Ecker et al., 2010b; Sato et al., 2013; Zheng et al., 2019). Jiao et al. (2010) constructed a small-scale dataset that contains 22 ASD and 16 normal control subjects (NC), and defined voxel-based and surface-based features. Four machine-learning techniques: support vector machines (SVMs), multilayer perceptrons (MLPs), functional trees (FTs), and logistic model trees (LMTs) were employed to classify ASD. LMT achieved the best accuracy of 87.0% for surface-based classification. Ecker et al. (2010a) proposed a five-dimensional feature followed by SVM to distinguish ASD from NC. It achieved the classification accuracy of 79.0% in the left hemisphere, 65.0% in the right on a single-site dataset. Although these methods reach a satisfactory diagnosis, the handcrafted features they used mainly come primarily from experience, also are bound by the experience.

Given the drawbacks of machine learning, some deep neural networks automatically acquire effective feature representation from sMRI data (Heinsfeld et al., 2018; Lian et al., 2018). However, the conflict between a small sample size and huge model parameters will lead to overfitting or other erratic model behavior. Thus, it is necessary to outline critical features from the MRI data before being fed into the networks. The morphological brain networks measuring the intracortical similarities in the gray matter play a crucial role in investigating abnormalities in neurological diseases (He et al., 2007; Yu et al., 2018).

Kong et al. (2019) proposed a simple individual brain network to express connectivity features between each pair of regions of interest (ROIs). Then the connectivity features are ranked by F-score in descending order. Finally, 3,000 top features were selected to perform classification *via* a DNN network. It achieved an accuracy of 90.39% in a subset of 182 subjects. However, it only carried out bi-level (ASD/TC) classification, neither was a large dataset from a multi-site included, nor the biomarker considered. To fix the problems, (Gao et al., 2021) used a Res-Net and Grad-CAM on individual structural covariance networks to perform the ASD diagnosis and biomarker identification. They achieved an accuracy of 71.8% on the ABIDE dataset and confirmed the prefrontal cortex and cerebellum as the biomarkers for ASD.

Though these methods achieved remarkable performance, they still have the following drawbacks: (1) The small sample size leads to overfitting and generalization problems, not to mention a small sample size from a single site. The single-site datasets can neither represent the variance of disease and control samples, nor establish stable generalization models for replication across different sites, participants, imaging parameters, and analysis methods (Nielsen et al., 2013). (2) So far, most machine learning methods for ASD diagnosis on sMRI data have considered morphological features extracted at different ROIs independently, ignoring the integrality of brain structure, and even in Gao et al. (2021), although the individual structural covariance networks are fed into the deep learning framework, the CNN framework only extracts the local feature by the kernel, which is not suitable for the brain network data. (3) The classification results from the deep learning model are hard to interpret in the absence of the contributions of the classification features leading to a lack of clinical significance. Although some biomarkers were found in Gao et al. (2021) by Grad-CAM (Selvaraju et al., 2017), it is fit for CNN-based models to produce the decisional explanations. The residual learning in Gao et al. (2021) is not suitable for the covariance networks, which leads to the biomarkers obtained from Grad-CAM being narrowly acceptable. Furthermore, there still exist gradient saturation and false confidence problems in Grad-CAM (Wang et al., 2020).

In view of the drawbacks, to explore an efficient ASD diagnosis method, we propose a self-attention deep learning framework to diagnose ASD and identify biomarkers on a multi-site dataset. This work is divided into two steps: first, we construct the individual morphological brain network from sMRI to characterize the interregional morphological relationship, and then, the output of morphological networks, instead of sMRI, is fed into a self-attention deep learning model to classify ASD from NCs. Meanwhile, the regional biomarkers are identified by the attention weight presenting the degree of contribution of the corresponding regional feature.

In the following sections, we will present our materials and methods in section “Materials and Methods,” results in section “Results,” discussion in Section “Discussion,” and conclusion in section “Conclusion.”

## Materials and Methods

### The Dataset

The ABIDE dataset (Di Martino et al., 2014), a large open access data repository, is used in this study, which is accessed from 17 international sites with no prior coordination. It includes structural MRI, corresponding rs-fMRI, and phenotype information for individuals with ASD and TC, which allows for replication, secondary analyses, and discovery efforts. Even if all data in it were collected with 3T scanners, the sequence parameters and the type of scanner varied across sites. In our work, the structural MR images we used were aggregated from all 17 international sites, which contain 518 ASD patients and 567 age-matched normal controls (ages 7–64 years, median 14.7 years across groups). In addition, the data we used contains 926 males and 159 females.

### Data Preprocessing

We used DRAMMS (Ou et al., 2011) to process all structural MR images in the preprocessing step, in which the cross-subject registration, motion correction, intensity normalization, and skull stripping are included. Furthermore, all T1W MRI images were registered to the SRI24 atlas (Rohlfing et al., 2010) for subsequent analysis. Then, We used the multiplicative intrinsic component optimization (MICO) method (Li et al., 2014) to segment the T1W images into the cerebrospinal fluid (CSF), white matter (WM), and gray matter (GM).

### Individual-Level Morphological Covariance Brain Networks

In our study, the individual level morphological covariance brain network (Wang et al., 2016) is used to extract interregional structural variations to characterize the interregional morphological relationship. The detailed procedures are described below. First, a GM volume map was acquired for each participant in the template space. Second, the individual-level morphological covariance brain network was constructed from their GM volume images, which refers to the literature (Wang et al., 2016). Although the SRI24 atlas parcellates the whole brain into 116 subregions, with 58 subregions in each hemisphere, due to the low signal-to-noise ratio and blank values of the gray matter volume in the Vermis, eight regions in the Vermis (the cerebellar Vermis labeled from 108 to 115) were excluded to ensure the reliability of our study. Finally, a 108× 108 matrix was obtained according to SRI24 atlas. That is, a vector *X*_*p*_ for each region and a matrix ***X*** for the whole brain were obtained for each subject for further analyses.

To be specific, the variation *x*_*pq*_is calculated as follows: the probability density function (PDF) of the extracted GM volume values is first estimated by the kernel density estimation (KDE) (Parzen, 1962).

Then, the variation of the KL divergence (KLD) between the region *P* and *Q* is calculated subsequently from the above PDFs as Eq. 1:


(1) $$D_{K\,L}\,\left(P,Q\right)=\sum_{i=1}^{N}\left(P\,\left(i\right)\,\log\frac{P\,\left(i\right)}{Q\,\left(i\right)}+Q\,\left(i\right)\,\log\frac{Q\,\left(i\right)}{P\,\left(i\right)}\right)$$

where _*P(i)*_ and _*Q(i)*_ are the PDFs of the region _*P*_ and _*Q*_. _*N*_ is the number of PDF sample points. The element of variation matrix is formally defined as the structural variation between two regions, which is quantified by a KL divergence-based similarity (KLS) measure (Kong et al., 2014) with the calculated variation of KLD. Thus, the variation between the region _*P*_ and _*Q*_ can be defined as Eq. 2:


(2) $$x_{P\,Q}=K\,L\,S\,\left(P,Q\right)={\text{e}}^{-D_{K\,L}\,\left(P,Q\right)}$$

Finally, the structural variation feature vector for the region *P* can be described as Eq. 3:


(3) $$X_{p}={\left(x_{p\,0},x_{p\,1},\ldots ,x_{p\,\left(M-1\right)}\right)}^{T}\in R^{M\times 1}$$

The structural variation matrix _*X*_ can be described as Eq. 4:


(4) $$X=\left(x_{p\,q}\right)=\left(X_{0},X_{1},\ldots ,X_{M-1}\right)\in R^{M\times M}$$

where _*M*_ is the number of regions, which is set as 108 in our study.

In the classification task, the matrix _*X*_ can be fed into the neural networks to replace the structural MR images.

### Self-Attention Neural Network Classifier

Transformer was first applied to machine translation tasks and has achieved great success in the field of natural language processing (Vaswani et al., 2017). The tremendous success in NLP has led researchers to adapt it to computer vision, where it has achieved great performance on the tasks of image classification (Dosovitskiy et al., 2020) and general-purpose backbone for computer vision (Liu et al., 2021). Especially, the transformer is designed for sequence modeling and transduction tasks, and the self-attention mechanism is notable for modeling long-range dependencies in the data (Wang et al., 2018; Cao et al., 2019; Liu et al., 2021). As the basis for powerful architectures, the self-attention mechanism in transformer has displaced CNN and RNN across a variety of tasks (Vaswani et al., 2017; Zhao et al., 2020; Han et al., 2021; Liu et al., 2021; Radford et al., 2021; Touvron et al., 2021; Wang et al., 2021; Yuan et al., 2021).

Morphological brain network refers to the intracortical variations in gray matter morphology. It is presented as the structural variation matrix among brain regions. In our work, the information of a brain region is represented by a feature vector to characterize its variation with other regions, and we expect to extract global information from the feature vectors of brain regions for the diagnosis. Thus, an optimal arrangement of data and feature extraction method are important for our work. Gao et al. (2021) viewed all region features as a matrix and fed it into a CNN framework. However, the CNN model only utilizes the local representation property of the extracted features by the convolution kernel; neither the dependency relationships between non-local regions are considered. The self-attention mechanism is adept at handling non-local dependencies in the data, which is able to take the place of the CNN model and extract the non-local feature from the data. Thus, it is suitable for the morphological brain network.

The output vectors of a self-attention layer are the weighted sum of input vectors, and the weight assigned to each vector is computed by the similarity of two vectors.

In the classification step, the vectors *X* = (*X*_0_,*X*_1_,…,*X*_*M*−1_) are first fed into the self-attention layer, and the query *Q*_*p*_, keys *K*_*p*_ and values *V*_*p*_ for the region *P* are defined as Eqs 5–7:


(5) $$Q_{p}=W_{Q}\,X_{p}$$

(6) $$K_{p}=W_{K}\,X_{p}$$

(7) $$V_{p}=W_{V}\,X_{p}$$

where *X_p_* is the variation feature vector for the region _*P*_, *W_Q_*, *W_K_*, and *W_V_* are the parameters to be learned.

Then, the self-attention coefficients _αpq_ are computed *via* dot product attention as Eq. 8:


(8) $$\alpha_{p\,q}=S\,o\,f\,t\,m\,a\,x\,\left(\frac{Q_{p}^{T}\,K_{q}}{\sqrt{d_{K}}}\right)$$

where *d_K_* is the dimension of *K_q_*.

Finally, the output vector $X_{p}^{1}$ of the region _*P*_ after the self-attention layer is computed as Eq. 9:


(9) $$X_{p}^{1}=\sum_{q=0}^{M-1}\alpha_{p\,q}\,V_{q}$$

### Biomarker Identification Based on Self-Attention Model

As the weight of the input feature vectors, the self-attention coefficients _α_ can indicate the contribution of the input vector to the output. Therefore, the self-attention coefficient map can be considered as the basis for the identification of biomarkers. The larger the weight _α_ of a feature vector is, the higher its contribution to the classification task is, and the more likely the corresponding brain region is the biomarker for ASD diagnosis.

### Implementation

**FIGURE 1:**
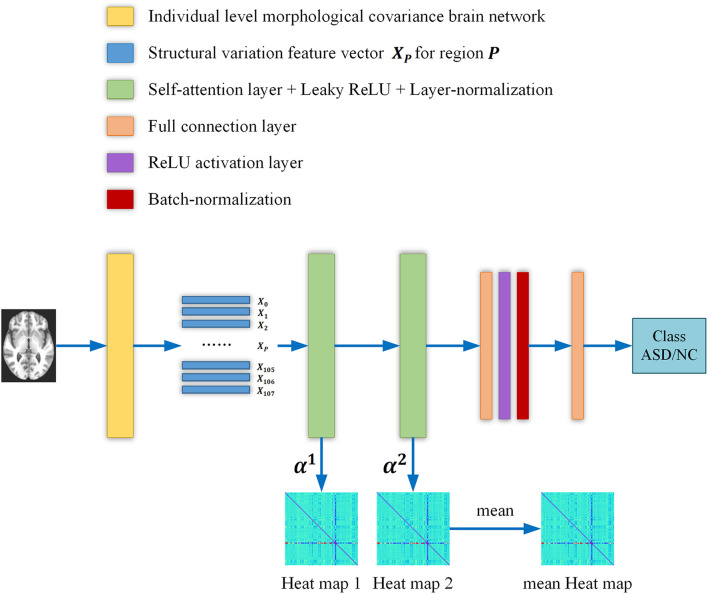
The overall flow chart of our study. Briefly, the individual level morphological covariance brain network is first constructed according to the SRI24 atlas and gray matter volume map of each subject. The above morphological covariance brain network is used to extract interregional structural variation vectors to characterize the interregional morphological relationship. Then the vectors are fed into two self-attention layers classification neural networks. Meanwhile, the contribution of each region for classification is obtained from the self-attention coefficients map of each layer. Finally, two heat maps are averaged to obtain a mean output heat map for diagnosis biomarker identification.

An overview of our proposed ASD/NC classification framework is shown in Figure 1, and two self-attention layers were adopted in the networks. First, we constructed an individual-level morphological covariance brain network according to the SRI24 atlas to obtain the structural variation feature vectors for each region. Then, the vectors were fed into two self-attention layers classification neural networks. In this work, the structural variation feature vector *_X_p_∈R_^108×1^* covers 108 regions, and the size of the output vectors $X_{p}^{1}$ and $X_{p}^{2}$ of each self-attention layer is _*R*_*^32×1^*. Meanwhile, the contribution of each region for classification was obtained from the self-attention coefficients map of each layer. After each self-attention layer, Leaky ReLU activation and layer-normalization (Ba et al., 2016) were adopted to ensure the training was stable and efficient. The negative slope of the Leaky ReLU activation layer is settled as _1e–2_, and the input feature size of each linear layer is _*R*_*^32×1^*. After the first linear layer, a ReLU activation and a batch-normalization (Ioffe and Szegedy, 2015) layer were adopted. We employed an Adam optimizer (Kingma and Ba, 2014) with the learning rate of _6e–6_. A batch size of 32 and a weight decay of 0.01 are used. After initializing the weights randomly, the binary cross-entropy loss is chosen to supervise the training for the ASD/NC classification.

## Results

**FIGURE 2:**
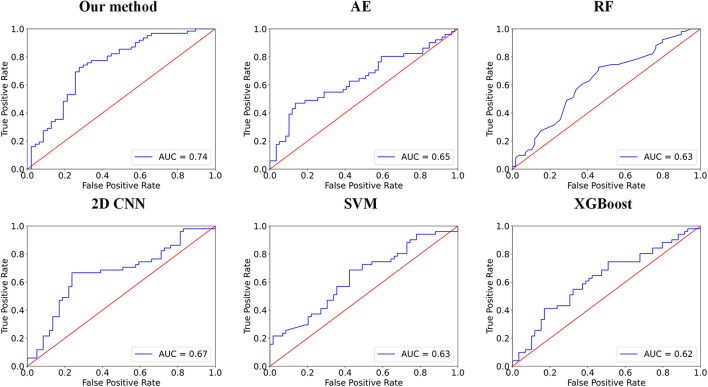
Comparisons between our method and other methods for classification. The area under the curve (AUC) values are used to assess the classification performances for our method, 2D CNN, autoencoder (AE), RF, support vector machine (SVM), and Xgboost (XGB).

In this group of experiments, we compare our framework with six competing methods in the task of ASD *versus* NC classification. Four parameters, namely accuracy (ACC), sensitivity (SEN), specificity (SPE), and F1 score, are calculated to evaluate the performance of our proposed framework. The deep self-attention neural networks used in our work achieved a mean classification accuracy of 72.5%, mean sensitivity value of 75.8%, specificity value of 68.1%, and F1 score of 0.758. Our results improved the mean classification accuracy of the state-of-the-art (Gao et al., 2021) from 71.8 to 72.5% in the ABIDE data. To evaluate the performance of our work, the result of our framework is compared with those of conventional machine learning methods [i.e., RF (Ho, 1995), SVM (Vapnik et al., 1998), and Xgboost (XGB) (Chen and Guestrin, 2016)] and deep learning methods [i.e., autoencoder (AE), 2D CNN (Gao et al., 2021) and 3D CNN]. Note that with the purpose of using structural variation matrix _*X∈R*_*^108×108^* for subject classification by these conventional machine learning methods and AE, it is first collapsed in a one-dimension vector _*Y∈R*_*^11664×1^*. Specifically, the dimension of the vector _*Y*_ was first reduced by Principal Component Analysis (PCA) in SVM classification, and the material sMRI images were fed into 3D CNN neural networks. The comparisons are presented in Table 1. Furthermore, the performance assessed by the area under the curve (AUC) values of these classifiers is shown in Figure 2. Our proposed framework has the best performances in classifying ASD from NC with the highest ACC, F1 score, and AUC values compared with the other methods.

**TABLE 1:** Comparison of the classification performances between our method and other methods.

**Method**	**Accuracy**	**Sensitivity**	**Specificity**	**F1 score**
Self-attention(ous)	**0.7248**	0.7581	0.6809	**0.7581**
RF	0.6091	0.4902	0.7119	0.5376
SVM	0.5818	0.3726	0.7627	0.4524
Xgboost	0.6091	0.5294	0.6780	0.5567
AE	0.6727	0.6875	**0.8750**	0.5714
2D CNN	0.7182	**0.8125**	0.6875	0.6869
3D CNN	0.5596	0.5714	0.4545	0.7000

*SVM, support vector machine; XGB, Xgboost; AE, autoencoder. The bold values indicate maximum value of each index.*

In our work, the self-attention layer can be set as a multi-head self-attention layer. Through comparison of the experiments in Table 2, we found that the network with a single-head self-attention layer achieved the best performance. There is the same number of parameters to be learned in the experiments in Table 2. In addition, through comparison presented in Table 3, we found that our model with two self-attention layers achieved the best performance.

**TABLE 2:** Comparison of the classification performances between the different number of heads in the self-attention layer.

**Method**	**Accuracy**	**Sensitivity**	**Specificity**	**F1 score**
1-Head	**0.7248**	**0.7581**	**0.6809**	**0.7581**
2-Head	0.6881	0.7549	0.6154	0.7167
4-Head	0.6789	0.7000	0.6410	0.7369
8-Head	0.6697	0.6667	0.6786	0.7500

*The bold values indicate maximum value of each index.*

**TABLE 3:** Comparison of our networks with the different number of self-attention layers.

**Number of Self- Attention Layers**	**Accuracy**	**Sensitivity**	**Specificity**	**F1 score**
1	0.6697	0.6627	**0.6923**	0.7534
2	**0.7248**	**0.7581**	0.6809	**0.7581**
3	0.6606	0.6711	0.6364	0.7338
4	0.6147	0.6667	0.5435	0.6667
5	0.6055	0.7174	0.5238	0.6055

*The bold values indicate maximum value of each index.*

**FIGURE 3:**
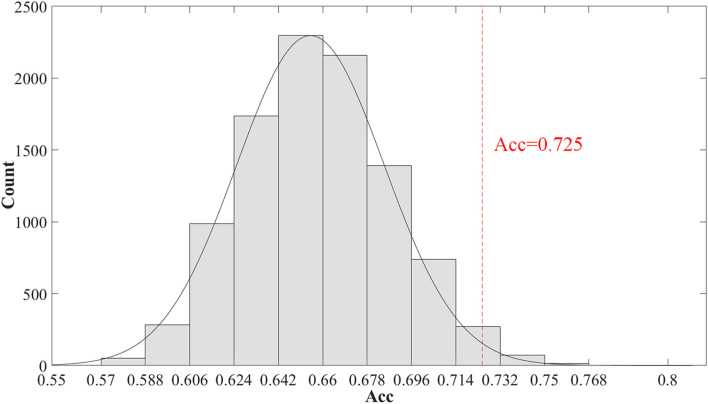
The histogram of the accuracy of the permutation test. The permutation test with 10,000 times was used to evaluate the significance of our method. The accuracy of our method (0.725) is indicated by the red dotted line. The classification accuracy is higher than 96.4% of the permutated accuracy values.

Furthermore, we evaluated the significance of the classification accuracy by the permutation test 10,000 times. During the permutation testing, we changed 20% of the labels of the samples randomly each time. The histogram of the accuracy of the permutation test is shown in Figure 3. The accuracy of our method (72.5%) is indicated by the red dotted line. As shown in Figure 3, the 72.5% accuracy of our method is higher than 96.4% of the permutated accuracy values.

**FIGURE 4:**
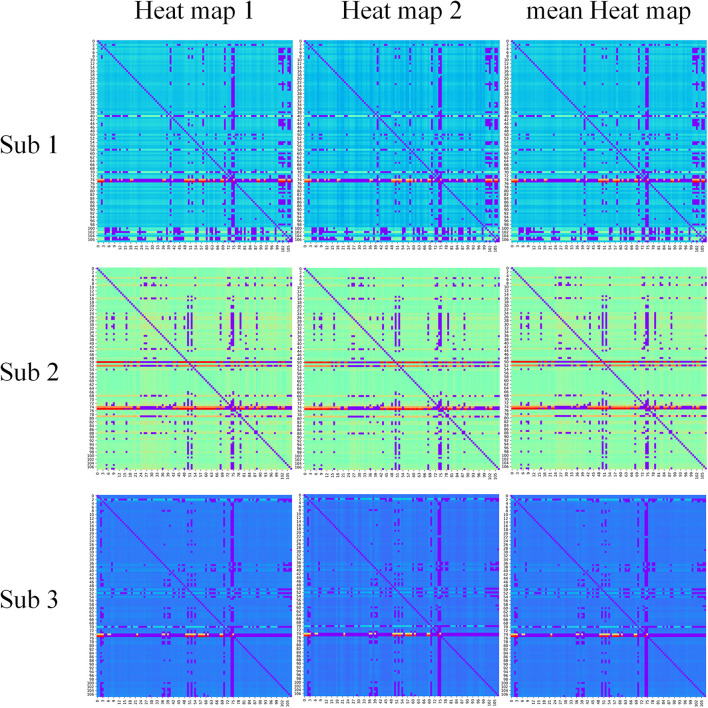
The contribution of brain regions to the autism spectrum disorder/normal control (ASD/NC) classification. Three typical individual heat maps supporting the correct classification of ASD patients were mapped by self-attention coefficients in our framework. The redder the color is, the more contributions the brain region affords.

**FIGURE 5:**
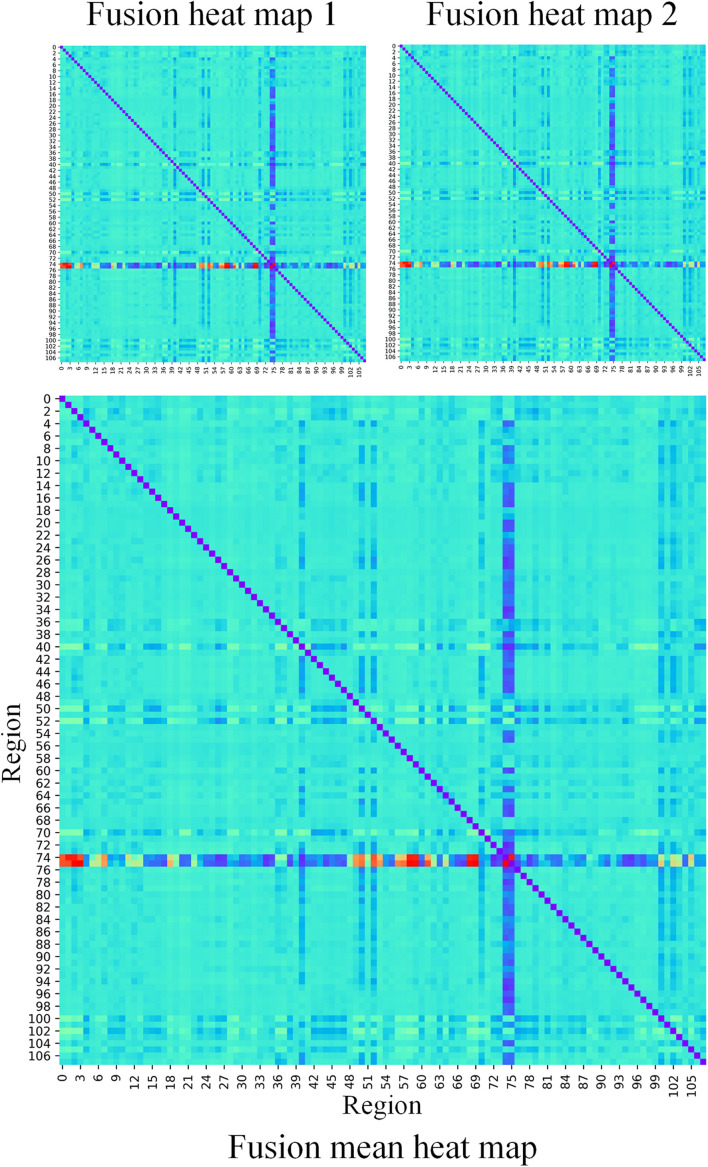
The fusion heat maps obtained by the average heat maps of all true negative subjects. The redder the color is, the more contributions the brain region affords.

**FIGURE 6:**
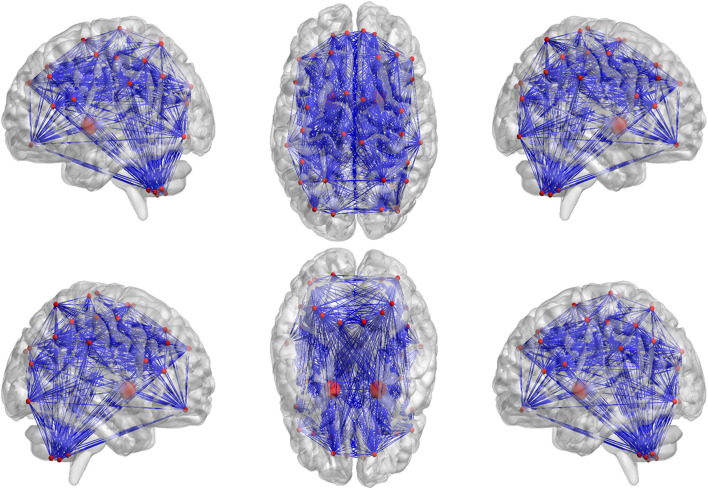
The top 42 regions which have the largest contributions to correctly classifying ASD patients were identified. As seen in our result, the pallidum in the left and right hemispheres have been found to be more significant and specific for ASD, which are drawn larger than other regions.

In our proposed framework, the self-attention coefficients _α_ were obtained through the self-attention layer, which can be seen as the contribution indicator of brain regions to the ASD/NC classification. In order to make our proposed model diagnose ASD more transparent and explainable, the self-attention coefficients map is obtained according to the following step. According to Eqs 8, 9, the self-attention coefficient _αpq_ indicates the contribution of the feature vector _*Xq*_ to the output feature vector $X_{p}^{1}$. Thus, the larger _αpq_ is, the larger the contribution of the region _*Q*_ to the classification is. In our result, we found that the self-attention coefficients maps of the first and second layers are extremely similar, so we average them to obtain a mean output coefficients map. In our work, the self-attention coefficients were first ranked in descending order. Then, the top coefficients were selected to determine the biomarker of regions. Three typical individual and final fused contributions supporting the correct classification of ASD patients are shown in Figures 4, 5. In these subfigures, the redder the regional color is, the more contribution the brain region affords. We selected the largest contributions of the regions by identifying the weights above the _mean+3 SD_. Finally, 53 coefficients about 42 different regions were found by the self-attention coefficients. The top 42 regions (see Figure 6) are significant for ASD/NC classification. Specifically, the feature vectors of these regions were selectively aggregated into the output feature vectors of two especial regions, which represent pallidum in the left and right hemispheres according to SRI24 atlas. It indicates that not only the 42 regions are significant for classification, but also the pallidum is more significant and specific. As seen in our result, the structure of pallidum has been found to be more significant than other regions for ASD, which is identical with the result in Turner et al. (2016).

## Discussion

In this manuscript, we propose a new framework for ASD detection and structural biomarkers identification from multi-site sMRI datasets by individual brain networks and self-attention deep neural networks. Our method has achieved state-of-the-art on ASD/NC classification task in the ABIDE data. Compared with the majority of machine learning and deep learning methods, our method has the following advantages: First, our work is stable and generalized due to the multi-site sMRI dataset with a large sample size, and the multi-site dataset is able to overcome the inherent heterogeneity in neuroimaging datasets.

Second, interregional structural variations can be extracted by the individual level morphological covariance network to characterize the interregional morphological relationship of the brain. Compared with the group-level morphological network, the individual-level morphological brain networks can better reflect individual behavior differences in both typical and atypical populations (Gao et al., 2021). Furthermore, the individual level morphological covariance network provides further empirical evidence to support the theory that the human brain has evolved to support both specialized or modular processing in local regions and distributed or integrated processing over the entire brain (Bullmore and Sporns, 2012; Wang et al., 2016). Thus, it provides an alternative method for researchers to explore hubs of the brain under both healthy and pathological conditions.

Third, the self-attention neural networks adopted in our model can aggregate not only short-range but also long-range dependencies in the data, which solves the local problem in CNN (Wang et al., 2018; Cao et al., 2019; Fu et al., 2019; Lee et al., 2019; Yin et al., 2020; Liu et al., 2021). Meanwhile, the biomarkers are obtained from self-attention coefficients without model architectural changes or retraining (Sarlin et al., 2020). Specifically, the heat maps of different layers obtained by Grad-CAM in Gao et al. (2021) have a clear hierarchical relationship, which is related to the feature extraction method of CNN. With the increase of the number of network layers, the receptive field becomes large, and the features extracted by CNN change from simple and local to complex and abstract (Wang et al., 2018). Therefore, the heat maps of different layers in Gao et al. (2021) vary greatly. However, the self-attention coefficients maps of the first and second layers in our method are extremely similar, which implies the consistency of the diagnosis. Furthermore, the diagnosis biomarker identification method based on self-attention coefficients is interpretable because the meaning of coefficients can be clearly obtained in Eqs 5–9 (Sarlin et al., 2020). In addition, due to the strong global feature extraction ability, the self-attention networks can achieve better performance than CNN with less training time and parameters in our work.

Moreover, with the self-attention explanation approach, the connectivity features of the morphological covariance network having the greatest contribution to classification were identified. The brain areas corresponding to these important connectivity features mainly include the prefrontal cortex, temporal cortex, and cerebellum. These brain areas have been reported to be implicated in ASD in previous studies indicating that the established classification model using deep learning and individual morphological covariance network may serve as a reliable tool to facilitate clinical diagnosis. For example, anatomically and functionally, there is considerable evidence that the medial prefrontal cortex is involved in basic conscious feelings, and the atypicality of it is associated with the emotional–social domain in autism (Shalom, 2009). The direct connections between the auditory association areas of the superior temporal gyrus with the medial temporal cortex have been demonstrated to underlie recognition memory for sounds (Muñoz-López et al., 2015). Furthermore, the cerebellum is not only involved in motor coordination but that it also intervenes in cognitive operations, emotion, memory, and language (Silveri and Misciagna, 2000). Thus, the prefrontal cortex, temporal cortex, and cerebellum may be related to social cognition processing in ASD.

## Conclusion

In this work, we propose a classification neural network for ASD detection and structural biomarkers identification from multi-site sMRI datasets, which is based on self-attention neural networks and individual-level morphological covariance brain networks. Comparison by experiments, we found that our proposed method outperformed other conventional machine learning and deep learning classification methods for the classification of ASD. Moreover, the biomarker identification method based on self-attention coefficients is efficient and interpretable, which provides a new solution to the black-box problems of deep learning, and prefrontal cortex, temporal cortex, and cerebellum found by this method provide a good reference for ASD diagnosis. Meanwhile, the morphological alterations in the pallidum in autism are worthy of the attention of researchers.

## Data Availability Statement

The datasets presented in this study can be found in online repositories. The names of the repository/repositories and accession number(s) can be found below: http://fcon_1000.projects.nitrc.org/indi/abide/abide_I.html.

## Ethics Statement

The studies involving human participants were reviewed and approved by Ethical approval was obtained from the St. James’s Hospital/AMNCH (ref: 2010/09/07) and the Linn Dara CAMHS Ethics Committees (ref: 2010/12/07). Written informed consent to participate in this study was provided by the participants’ legal guardian/next of kin.

## Author Contributions

All authors contributed to the article and approved the submitted version.

## Conflict of Interest

The authors declare that the research was conducted in the absence of any commercial or financial relationships that could be construed as a potential conflict of interest.

## Publisher’s Note

All claims expressed in this article are solely those of the authors and do not necessarily represent those of their affiliated organizations, or those of the publisher, the editors and the reviewers. Any product that may be evaluated in this article, or claim that may be made by its manufacturer, is not guaranteed or endorsed by the publisher.

## References

[B1] Ba J. L. Kiros J. R. Hinton G. E. (2016). Layer normalization. *arXiv* [preprint]. arXiv:06450,

[B2] Bullmore E. Sporns O. (2012). The economy of brain network organization. *Nat. Rev. Neurosci.* 13 336–349. 10.1038/nrn3214 22498897

[B3] Cao Y. Xu J. Lin S. Wei F. Hu H. (2019). “Gcnet: non-local networks meet squeeze-excitation networks and beyond,” in *Proceedings of the IEEE/CVF International Conference on Computer Vision Workshops.* 10.1109/ICCVW.2019.00246

[B4] Chen T. Guestrin C. (2016). “Xgboost: a scalable tree boosting system,” in *Proceedings of the 22nd Acm Sigkdd International Conference on Knowledge Discovery and Data Mining.* 10.1145/2939672.2939785

[B5] Di Martino A. Yan C.-G. Li Q. Denio E. Castellanos F. X. Alaerts K. (2014). The autism brain imaging data exchange: towards a large-scale evaluation of the intrinsic brain architecture in autism. *Mol. Psychiatry* 19 659–667. 10.1038/mp.2013.78 23774715 PMC4162310

[B6] Dosovitskiy A. Beyer L. Kolesnikov A. Weissenborn D. Zhai X. Unterthiner T. (2020). “An image is worth 16x16 words: transformers for image recognition at scale,” in *Proceeding of the International Conference on Learning Representations.*

[B7] Ecker C. Rocha-Rego V. Johnston P. Mourao-Miranda J. Marquand A. Daly E. M. (2010b). Investigating the predictive value of whole-brain structural MR scans in autism: a pattern classification approach. *Neuroimage* 49 44–56. 10.1016/j.neuroimage.2009.08.024 19683584

[B8] Ecker C. Marquand A. Mourão-Miranda J. Johnston P. Daly E. M. Brammer M. J. (2010a). Describing the brain in autism in five dimensions—magnetic resonance imaging-assisted diagnosis of autism spectrum disorder using a multiparameter classification approach. *J. Neurosci.* 30 10612–10623. 10.1523/JNEUROSCI.5413-09.2010 20702694 PMC6634684

[B9] Fu J. Liu J. Tian H. Li Y. Bao Y. Fang Z. (2019). “Dual attention network for scene segmentation,” in *Proceedings of the IEEE/CVF Conference on Computer Vision and Pattern Recognition.* 10.1109/CVPR.2019.00326

[B10] Gao J. Chen M. Li Y. Gao Y. Li Y. Cai S. (2021). Multisite autism spectrum disorder classification using convolutional neural network classifier and individual morphological brain networks. *Front. Neurosci.* 14:1473. 10.3389/fnins.2020.629630 PMC7877487 33584183

[B11] Guze S. B. (1995). Diagnostic and statistical manual of mental disorders, (DSM-IV). *Am. J. Psychiatry* 152 1228–1228. 10.1176/ajp.152.8.1228

[B12] Han K. Xiao A. Wu E. Guo J. Xu C. Wang Y. (2021). Transformer in transformer. *arXiv* [preprint]. arXiv:00112,

[B13] He Y. Chen Z. J. Evans A. C. (2007). Small-world anatomical networks in the human brain revealed by cortical thickness from MRI. *Cerebral Cortex* 17 2407–2419. 10.1093/cercor/bhl149 17204824

[B14] Heinsfeld A. S. Franco A. R. Craddock R. C. Buchweitz A. Meneguzzi F. (2018). Identification of autism spectrum disorder using deep learning and the ABIDE dataset. *NeuroImage Clin.* 17 16–23. 10.1016/j.nicl.2017.08.017 29034163 PMC5635344

[B15] Ho T. K. (1995). “Random decision forests,” in *Proceedings of 3rd International Conference on Document Analysis and Recognition*, (IEEE).

[B16] Ioffe S. Szegedy C. (2015). “Batch normalization: accelerating deep network training by reducing internal covariate shift,” in *proceeding of the International Conference on Machine Learning.*

[B17] Jiao Y. Chen R. Ke X. Chu K. Lu Z. Herskovits E. H. (2010). Predictive models of autism spectrum disorder based on brain regional cortical thickness. *Neuroimage* 50 589–599. 10.1016/j.neuroimage.2009.12.047 20026220 PMC2823830

[B18] Kingma D. P. Ba J. (2014). Adam: a method for stochastic optimization. *arXiv* [preprint]. arXiv:1412.6980,

[B19] Kong X.-Z. Wang X. Huang L. Pu Y. Yang Z. Dang X. (2014). Measuring individual morphological relationship of cortical regions. *J. Neurosci. Methods* 237 103–107. 10.1016/j.jneumeth.2014.09.003 25220868

[B20] Kong Y. Gao J. Xu Y. Pan Y. Wang J. Liu J. (2019). Classification of autism spectrum disorder by combining brain connectivity and deep neural network classifier. *Neurocomputing* 324 63–68. 10.1016/j.neucom.2018.04.080

[B21] Lee J. Lee Y. Kim J. Kosiorek A. Choi S. Teh Y. W. (2019). “Set transformer: a framework for attention-based permutation-invariant neural networks,” in *Proceeding of the International Conference on Machine Learning.*

[B22] Li C. Gore J. C. Davatzikos C. (2014). Multiplicative intrinsic component optimization (MICO) for MRI bias field estimation and tissue segmentation. *Magnetic Resonance Imaging* 32 913–923. 10.1016/j.mri.2014.03.010 24928302 PMC4401088

[B23] Lian C. Liu M. Zhang J. Shen D. (2018). Hierarchical fully convolutional network for joint atrophy localization and Alzheimer’s disease diagnosis using structural MRI. *IEEE Trans. Pattern Analy. Mach. Intelli.* 42 880–893. 10.1109/TPAMI.2018.2889096 30582529 PMC6588512

[B24] Liu Z. Lin Y. Cao Y. Hu H. Wei Y. Zhang Z. (2021). Swin transformer: hierarchical vision transformer using shifted windows. *arXiv* [preprint]. arXiv:14030,

[B25] Muñoz-López M. Insausti R. Mohedano-Moriano A. Mishkin M. Saunders R. (2015). Anatomical pathways for auditory memory II: information from rostral superior temporal gyrus to dorsolateral temporal pole and medial temporal cortex. *Front. Neurosci.* 9:158. 10.3389/fnins.2015.00158 26041980 PMC4435056

[B26] Nielsen J. A. Zielinski B. A. Fletcher P. T. Alexander A. L. Lange N. Bigler E. D. (2013). Multisite functional connectivity MRI classification of autism: ABIDE results. *Front. Hum. Neurosci.* 7:599. 10.3389/fnhum.2013.00599 24093016 PMC3782703

[B27] Ou Y. Sotiras A. Paragios N. Davatzikos C. (2011). DRAMMS: deformable registration *via* attribute matching and mutual-saliency weighting. *Med. Image Analy.* 15 622–639. 10.1016/j.media.2010.07.002 20688559 PMC3012150

[B28] Parzen E. (1962). On estimation of a probability density function and mode. *Ann. Math. Statist.* 33 1065–1076. 10.1214/aoms/1177704472

[B29] Radford A. Kim J. W. Hallacy C. Ramesh A. Goh G. Agarwal S. (2021). Learning transferable visual models from natural language supervision. *arXiv* [preprint]. arXiv:00020,

[B30] Rohlfing T. Zahr N. M. Sullivan E. V. Pfefferbaum A. (2010). The SRI24 multichannel atlas of normal adult human brain structure. *Hum. Brain Mapp.* 31 798–819. 10.1002/hbm.20906 20017133 PMC2915788

[B31] Sarlin P.-E. DeTone D. Malisiewicz T. Rabinovich A. (2020). “Superglue: learning feature matching with graph neural networks,” in *Proceedings of the IEEE/CVF Conference on Computer Vision and Pattern Recognition.* 10.1109/CVPR42600.2020.00499

[B32] Sato J. R. Hoexter M. Q. de Magalhães Oliveira P. P. Jr. Brammer M. J. Murphy D. Ecker C. (2013). Inter-regional cortical thickness correlations are associated with autistic symptoms: a machine-learning approach. *J. Psychiatric Res.* 47 453–459. 10.1016/j.jpsychires.2012.11.017 23260170

[B33] Selvaraju R. R. Cogswell M. Das A. Vedantam R. Parikh D. Batra D. (2017). “Grad-cam: visual explanations from deep networks *via* gradient-based localization,” in *Proceedings of the IEEE International Conference on Computer Vision.*

[B34] Shalom D. B. (2009). The medial prefrontal cortex and integration in autism. *Neuroscientist* 15 589–598. 10.1177/1073858409336371 19617590

[B35] Silveri M. C. Misciagna S. (2000). Language, memory, and the cerebellum. *J. Neurolinguistics* 13 129–143. 10.1016/S0911-6044(00)00008-7

[B36] Touvron H. Cord M. Douze M. Massa F. Sablayrolles A. Jégou H. (2021). “Training data-efficient image transformers & distillation through attention,” in *Proceeding of the International Conference on Machine Learning.*

[B37] Turner A. H. Greenspan K. S. van Erp T. G. (2016). Pallidum and lateral ventricle volume enlargement in autism spectrum disorder. *Psychiatry Res. Neuroimag.* 252 40–45. 10.1016/j.pscychresns.2016.04.003 27179315 PMC5920514

[B38] Vapnik V. Suykens J. A. K. Vandewalle J. (eds) (1998). “The support vector method of function estimation,” in *Nonlinear Modeling*, (Boston, MA: Springer), 55–85. 10.1007/978-1-4615-5703-6_3

[B39] Vaswani A. Shazeer N. Parmar N. Uszkoreit J. Jones L. Gomez A. N. (2017). “Attention is all you need,” in *Proceedings of the Advances in Neural Information Processing Systems.First 12 Conferences.*

[B40] Wang H. Jin X. Zhang Y. Wang J. (2016). Single-subject morphological brain networks: connectivity mapping, topological characterization and test–retest reliability. *Brain Behav.* 6:e00448. 10.1002/brb3.448 27088054 PMC4782249

[B41] Wang H. Wang Z. Du M. Yang F. Zhang Z. Ding S. (2020). “Score-CAM: score-weighted visual explanations for convolutional neural networks,” in *Proceedings of the IEEE/CVF Conference on Computer Vision and Pattern Recognition Workshops.* 10.1109/CVPRW50498.2020.00020

[B42] Wang W. Xie E. Li X. Fan D.-P. Song K. Liang D. (2021). Pyramid vision transformer: a versatile backbone for dense prediction without convolutions. *arXiv* [preprint]. arXiv:12122,

[B43] Wang X. Girshick R. Gupta A. He K. (2018). “Non-local neural networks,” in *Proceedings of the IEEE Conference on Computer Vision and Pattern Recognition.* 10.1109/CVPR.2018.00813

[B44] Yin M. Yao Z. Cao Y. Li X. Zhang Z. Lin S. (2020). “Disentangled non-local neural networks,” in *European Conference on Computer Vision*, eds Vedaldi A. Bischof H. Brox T. Frahm J. M. (Cham: Springer). 10.1007/978-3-030-58555-6_12

[B45] Yu K. Wang X. Li Q. Zhang X. Li X. Li S. (2018). Individual morphological brain network construction based on multivariate euclidean distances between brain regions. *Front. Hum. Neurosci.* 12:204. 10.3389/fnhum.2018.00204 29887798 PMC5981802

[B46] Yuan L. Chen Y. Wang T. Yu W. Shi Y. Jiang Z. (2021). Tokens-to-token vit: training vision transformers from scratch on imagenet. *arXiv* [preprint]. arXiv:11986,

[B47] Zhao H. Jia J. Koltun V. (2020). “Exploring self-attention for image recognition,” in *Proceedings of the IEEE/CVF Conference on Computer Vision and Pattern Recognition.* 10.1109/CVPR42600.2020.01009

[B48] Zheng W. Eilamstock T. Wu T. Spagna A. Chen C. Hu B. (2019). *Multi-Feature Based Network Revealing the Structural Abnormalities in Autism Spectrum Disorder.* Piscataway: IEEE Transactions on Affective Computing.

